# SIRT 3 was involved in *Lycium barbarum* seed oil protection testis from oxidative stress: *in vitro* and *in vivo* analyses

**DOI:** 10.1080/13880209.2021.1961822

**Published:** 2021-09-26

**Authors:** Zhang-Jie Yang, Yu-Xin Wang, Shuai Zhao, Na Hu, Dong-Mei Chen, Hui-Ming Ma

**Affiliations:** a Key Laboratory of Fertility Preservation and Maintenance of Ministry of Education in Ningxia Medical University, Yinchuan, China; b Institute of Human Stem Cell Research, The General Hospital of Ningxia Medical University, Yinchuan, China; c College of Chinese medicine of Ningxia Medical University, Yinchuan, China

**Keywords:** Sertoli cells (TM4), anti-ageing, ethic medicine

## Abstract

**Context:**

*Lycium barbarum* L. (Solanaceae) seed oil (LBSO) exerts LBSO exerts protective effects in the testis *in vivo* and *in vitro* via upregulating SIRT3.

**Objective:**

This study evaluates the effects and mechanism of LBSO in the d-galactose (d-gal)-induced ageing testis.

**Materials and methods:**

Male Sprague Dawley (SD) rats (*n* = 30, 8-week-old) were randomly divided into three groups: LBSO group (*n* = 10) where rats received subcutaneous injection of d-gal at 125 mg/kg/day for 8 weeks and intragastric administration of LBSO at 1000 mg/kg/day for 4 weeks, ageing model group (*n* = 10) received 8-week-sunbcutaneous injection of d-gal, and control group (*n* = 10) with same administration of normal saline. Lentivirus had established TM4 cells with SIRT3 overexpression or silencing before LBSO intervened *in vitro*.

**Results:**

Treatment with LBSO, the levels of INHB and testosterone both increased, compared to ageing model. *In vitro*, we found the ED_50_ of LBSO was 86.72 ± 1.49 and when the concentration of LBSO at 100 μg/mL to intervene TM4 cells, the number of cells increased from 8120 ± 676.2 to 15251 ± 1119, and the expression of SIRT3, HO-1, and SOD upregulated. However, HO-1 and SOD were dysregulated by silencing SIRT3. On the other hand, the expression of AMPK and PGC-1*α* upregulated as an effect of SIRT3 overexpression by lentivirus, meanwhile the same increasing trend of that being found in cells treated with LBSO, compared to control group.

**Discussion and conclusions:**

LBSO alleviated oxidative stress in d-gal-induced sub-acutely ageing testis and TM4 cells by suppressing the oxidative stress to mitochondria via SIRT3/AMPK/PGC-1α.

## Introduction

Ageing is commonly defined as the slow progressive functional decline of organisms with time, which is caused by the simultaneous deterioration of various interconnected cellular functions accompanied by ageing-related diseases that lower the quality of life (Kritsilis et al. 2018). The population of the world is rapidly ageing, and anti-ageing has gradually become an issue of global concern (Beard et al. 2017; Cosco et al. 2017), therefore it simplifies the anti-ageing treatments is especially important for everyone. Reportedly, in males, chronic oxidative damage to testicular tissue has a severe negative impact on male fertility (Leisegang et al. 2017) with the decrease in pituitary-gonad axis response, leading to the decline of testicular function and failure to preserve fertility and sexual function (Angelopoulou et al. 2013). Therefore, it is necessary to alleviate testicular oxidative injury in order to preserve testicular function in older men. The classical theory of ageing, put forth by Harman in 1956, stated that the mammalian lifespan depends on the genetic regulation of oxygen utilisation rate. Supposedly, the correlation between oxidative stress and ageing course has been tightly linked in several studies (Shaw et al. 2014).

In testicular tissue, Sertoli cells are composed of blood-testis-barrier (BTB) with Sertoli cell–cell and Sertoli germ cell junctions, where vital functions in spermatogenesis are effectuated (Ahmed et al. 2018; Luca et al. 2018a). Furthermore, Sertoli cells are not only a scaffold-like structural system but a dynamic functional system of intercellular support that delivers potent immunomodulatory (Luca et al. 2018b) and trophic factors (Tarulli et al. 2012; Crisóstomo et al. 2018). However, Sertoli cells are vulnerable to external stimulation that exerts internal changes and cause mitochondria impairment, leading to BTB disruption and spermatogenesis dysfunction (Cheng and Mruk 2012). Reportedly, oxidative stress in testis causes mitochondrial injury in Sertoli cells by the accumulation of reactive oxygen species (ROS) (Almeida et al. 2017). The cells of testes contain abundant mitochondria that maintain the function of testes, provide energy to the actions of cells but are vulnerable to oxidative substances, and hence, it is vital to clear away the redundancy of oxygen free radicals generated due to the external stimulation in testes (Zhao et al. 2019). Interestingly, in the ageing process, oxidative stress is the modulator of silent information regulator of transcription 3 (SIRT3) (Zhang et al. 2015). An earlier study has shown that SIRT3 is the main mitochondrial sirtuin involved in the regulation of mitochondrial oxidative stress, and the elimination of oxygen free radicals protects the individuals from ageing (Masaki 2010). SIRT3 is the main mitochondrial sirtuin involved in regulating key enzymes in oxidative phosphorylation via deacetylation, thereby controlling vigorous metabolism (Wang et al. 2019) in cardiomyocytes, hepatocytes, kidney cells, and testes (Liu et al. 2017). SIRT3 plays a critical role in the pathogenesis of DOX-induced cardiotoxicity (Yang et al. 2019) with functions such as relieving antioxidative stress (Sun et al. 2018), regulating apoptosis (Qiao et al. 2016), and participating in energy metabolism (Nogueiras et al. 2012). Furthermore, melatonin-induced oxidative stress via inhibition of the SIRT3/SOD2-AKT pathway in human histiocytic lymphoma (Li et al. 2020) plays a critical role in chronic Chagas disease by targeting the AMPK/NFE2L2/SIRT3 signalling pathway (Caballero et al. 2020). In addition to these effects, SIRT3 is also known as a human longevity protein (Albani et al. 2014). In SIRT3 knockout mice, diseases that commonly accompany ageing, especially cancer, exhibit accelerated processes (Ahn et al. 2008). Recent studies have proven that SIRT3 regulates mitochondrial oxidation reactions and reduces ROS production in cells (Xie et al. 2017). Therefore, SIRT3 may act as an endogenous protective factor and a new therapeutic target in ageing process.

*Lycium barbarum* L. (Solanaceae) is a traditional food and Chinese medicine that has nourished the liver and kidney (Tan et al. 2019) and has maintained fertility for thousands of years (Ren et al. 2019). Traditionally, *Lycium barbarum* seed oil (LBSO) is extracted from *Lycium barbaru*m seeds, cultured in the northwest of China, and widely used as a functional food (Potterat 2010). Several studies have shown that grape seed oil delays senescence by attenuating oxidative (Harbeoui et al. 2019) and inflammatory responses (Millan-Linares et al. 2018). Other oils, such as rice bran oil (Lee et al. 2019), olive oil (Perrone et al. 2019), and sunflower oil (Navarro-Hortal et al. 2019), also achieve a similar effect in anti-ageing. Therefore, the present study investigated the active functions on the antioxidative stress of LBSO and showed the effects of *L. barbarum* on protecting individuals from ageing by antioxidation and anti-inflammation; however, the active components of *L. barbarum* are yet elusive (Gao et al. 2017). Thus, this study investigated the effect of LBSO on antioxidative stress and illuminated the potential mechanism that might activate SIRT3.

In this study, we investigated the ability of LBSO to augment the antioxidative stress in the d-gal-induced ageing testis *in vivo* and ageing Sertoli cells (TM4) *in vitro*. Also, the mechanism of the effects of LBSO was explored on the anti-oxidation of mitochondria.

## Materials and methods

### Materials

LBSO was procured from Ningxia Qiming Biological Food Co., Ltd (Executive Standard: Q/QMSW0001S). d-Gal (> 99% pure) was purchased from Sigma-Aldrich (St. Louis, MO, USA). The antibodies to p-AMPK, p-PGC-1α, PGC-1α, and p-*γ*H2AX were purchased from Cell Signalling Technology (Boston, MA, USA). The β-galactosidase kits were bought from Boster (Wuhan, China). The antibodies to AMPK, SOD-1, and SOD-2 were purchased from Bioss (Beijing, China), the antibodies to SIRT3, p21^Waf1/Cip1^ and p16^INK4A^ were obtained from Wanleibio (Shenyang, China), and antibodies to β-galactosidase, NRF2, Wilms’ tumour (WT1), and HO-1 were bought from Abcam (Cambridge, UK). H_2_O_2_ inhibitor, HRP-conjugated secondary antibodies, DAB (3,3-diaminobenzidine), and anti-goat serum antibody were purchased from Bioss. The fluorescein and rhodamine-conjugated goat anti-mouse/rabbit IgG antibodies were purchased from Proteintech (Chicago, IL, USA). The chemiluminescence reagents were obtained from Wanleibio (Shenyang, China).

### Ethics statement

Sprague-Dawley (SD) male rats, purchased from the Laboratory Animal Centre of Ningxia Medical University, were fed in a specific pathogen-free (SPF) level laboratory (temperature: 20-26 °C; relative humidity: 40–50%). The experiments were approved by the Institutional Animal Care and Use Committee of the Ningxia Hui Autonomous Region (IACUC No. SCXK (Ning) 2015-0001 and NYLAC-2018-091).

### Animal experiments

Male SD rats (*n* = 30, 8-week-old), with initial body weight 230–255 g, were randomly divided into three groups: control, ageing model, and LBSO. The ageing model and LBSO groups received subcutaneous injection of d-gal at 125 mg/kg/day for 8 weeks, and the final concentration of d-gal was found to have achieved after examining the data from numerous studies (Huang et al. 2016; Liu et al. 2017). An equivalent volume of normal saline was administered to the control group. While subcutaneous injection continued for another 4 weeks, rats in the LBSO group received intragastric administration of LBSO at 1000 mg/kg/day according to the body weight; this dose proved effective in, as described in a previous study (Farcas et al. 2019; Yao et al. 2019). Moreover, the same volume of normal saline was administered intragastrically to the control and the ageing model for another 4 weeks. On the day of termination, the rats fasted overnight before dissection. Subsequently, the rats were anaesthetised by an intravenous injection of 0.2 mL/100 g of 2% pentobarbital sodium. After blood samples were withdrawn from the heart, the rats were euthanized by CO_2_ overdose. The serum was obtained by centrifugation of the blood at 3000 ×*g* at 4 °C for 10 min. The bilateral testes were perfectly dissected; one was fixed with 4% paraformaldehyde for histology analysis, and the other was stored at −80 °C for Western blot.

### Enzyme-linked immunosorbent assay (ELISA)

The serum separated by centrifugation (3000 ×*g* for 10 min at 4 °C), was evaluated using the ELISA kits (Cloud-Clone Co., Wuhan, China), according to the manufacturer’s instructions. The absorbance was measured at 450 nm on a micrometer (Thermo Scientific, Waltham, Massachusetts, USA).

### Histological analysis

Testicular tissue was fixed with 4% paraformaldehyde for 12 h. The sections were resected at 5 μm thickness. Haematoxylin-eosin (HE) staining: All slides were dewaxed and stained with eosin for 5 s before soaking in haematoxylin for 1 min. Immunohistochemistry (IHC): The slides were incubated for 12 h at 4 °C with primary antibody to β-galactosidase (1:100), followed by incubation with a secondary antibody for 20 min. The immunostained sections were developed by DAB and haematoxylin staining. The images were captured under a microscope (Leica, DM500, Solms, Germany). The expression of proteins was quantitated using Image Pro Plus 6.0 to compute the positive staining-integral optical density/area (IOD/area, density mean).

### Immunofluorescence (IF)

After dewaxing, 0.5% Triton X-100 was used to permeabilize the membrane for 20 min that was then blocked with the goat serum for 1 h at room temperature. The fluorescein or rhodamine-conjugated goat anti-mouse/rabbit IgG was applied for 1 h after the primary antibodies (SIRT3, 1:200; WTI,1:200) were added overnight at 4 °C. Subsequently, the tissues were disposed with DAPI for 5 min before observation under a laser confocal microscope (Nikon, Japan).

### Cells culture

Sertoli cells (TM4 cells) of mice were purchased from Shanghai Institute of Cell Science, Chinese Academy of Sciences and cultured in DMEM with 10% heat-inactivated foetal bovine serum [FBS (Gibco, Carlsbad, USA)] with 1% penicillin-streptomycin combination in the atmosphere of 95% humidity and 5% CO_2_ at 37 °C.

### Pre-treatment of LBSO in TM4 cells

Based on the ageing model established in TM4 cells, another ageing model widely applied in the scientific community was induced by 200 mmol/L d-gal for 48 h (Heidari et al. 2018). Then, the cells were cultured in 96-well plates and treated with LBSO (25, 50, 100, and 150 μg/mL, respectively) (Assmann et al. 2018).

### β-Galactosidase staining in cells

A total of 10000 TM4 cells/well were cultured in a 6-well plate and treated as described above. According to the instructions of β-galactosidase staining kit, the cells were fixed, and the galactosidase staining solution was added and incubated at 37 °C overnight. The blue staining was observed under an inverted microscope, indicating cell senescence. The positive staining rate was calculated by the number of stained cells in blue/number of whole cells × 100%.

### Lentivirus transfection

Lentivirus packaging and transfection were employed to establish cell lines with SIRT3 overexpression or silencing; the virus was purchased from GenePharma, China. The sequence of overexpression is 5′-GGGGCCGGCATCAGCACACCCAGTGGCATCCCGGACTTCAGATCCCCAGGGAGCGGCCTC-3′, and the siRNA sequence is 5′-GCGGCTCTATACACAGAACATCTCGAGATGTTCTGTGTATAGAGCCGC-3′.

A total of 3–5 × 10^5^ TM4 cells were cultured in the 6-well plate to 60–70% confluency and transferred by the packaged lentivirus diluted to 1:10 (titer: 2 × 10^8^ TU/mL). The transfected cells were cultured for an additional 6 h, following which, the culture medium was changed. After transfection for 48 h, the transfection efficiency was observed under a fluorescence microscope (Mshot, Guangzhou, China), and the ratio of the number of cells expressing green fluorescence infields to the total number of cells was calculated as the positive rate of transfection.

### Cell counting kit (CCK)-8 assay

An equivalent of 10,000 cells/well was cultured in 96-well plates. Post-treatment, 10 μL CCK-8 solution (Meilunbio Dalian China) was added to 100 μL medium into each well. The absorbance was measured at 450 nm on a micrometer (Thermo Scientific, Waltham, MA, USA).

### Western blotting

Excised testicular tissue, stored at −80 °C, was homogenised by ultrasonic equipment with protein lysis buffer (KeyGEN, Jiangsu, China) and incubated on ice for 10 min. The processed samples were separated by Sodium dodecyl sulphate polyacrylamide gel electrophoresis (SDS-PAGE) and transferred to Polyvinylidene fluoride (PVDF) membranes. Then, the membranes were blocked by 3% bovine serum albumin (BSA) for 1 h at room temperature and probed with primary antibodies (SIRT3, 1:500; NRF2, 1:300; HO-1, 1:800; SOD-1, 1:300; SOD-2, 1:300; AMPK,1:500; and p-AMPK, 1:500) overnight at 4 °C, followed by incubation with HRP-conjugated secondary antibody (1:15,000) for 1 h. Subsequently, the membranes were developed using chemiluminescence reagents.

### Statistical analysis

SPSS v20.0 software was used for statistical analysis. The data of Western blot and ELISA were quantified as the mean ± SD. One-way analysis of variance (ANOVA) was applied in more than three groups. SNK-q test was used to compare the mean values of the two groups of samples. The rates were analysed using the rank test, and *p* < 0.05 indicated statistical significance.

## Results

### Evaluation of ageing in testicular tissue of d-gal-induced ageing rats

**Figure 1.**
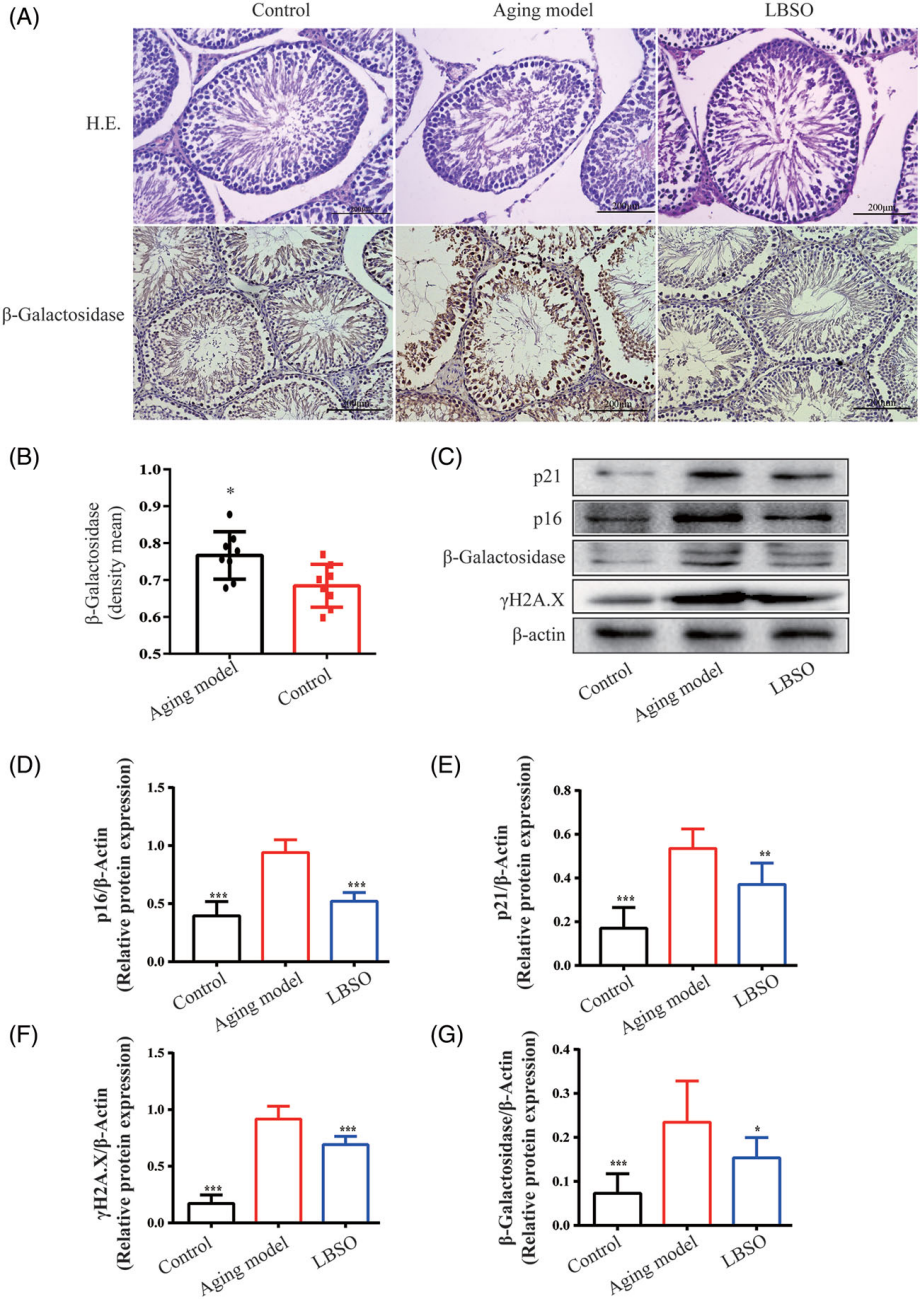
Effects of LBSO on anti-ageing in the testis in *vivo*. (A) Haematoxylin and eosin (H&E)-stained testicular tissues, 400× magnification. The expression of β-galactosidase located in the testicular tissue was observed by immunohistochemical staining (magnification 400×). (B) Analysis of the density mean of β-galactosidase. The data of the positive staining-integral optical density/area (IOD/area, density mean) were expressed as the mean ± SD, *n* = 10; *p < 0.05, compared to the control group. (C) The expression of p16^INK4A^, p21^Waf1/Cip1^, p-*γ*H2AX, and β-galactosidase in the testicular tissue was detected by Western blot. The relative expression of (D) p16^INK4A^, (E) p21^Waf1/Cip1^, (F) p-*γ*H2AX, and (G) β-galactosidase in testicular tissue. Densitometry was used to compare the expression levels. β-Actin was used as an internal loading control. All data were expressed as the mean ± SD, *n* = 10; *p < 0.05, **p < 0.01, ***p < 0.001, compared to the ageing model.

The current study was based on a rat model of sub-acute ageing via subcutaneous injection of d-gal for 8 weeks; differences were observed in the testicular histomorphology between the ageing and control groups. In Figure 1(A), HE staining showed that compared to the control group, a large number of spermatogenic epithelial germ cells were shed in the ageing model group, which significantly decreased the number of supporting cells and germ cells; also, the middle of convoluted seminiferous ducts was distorted. Moreover, β-galactosidase located in the ageing tissue was expressed markedly in the testicular tissue of ageing model rats, as assessed by IHC. The positive staining-integral optical density/area in ageing testicular tissue increased significantly as compared to the control group (*p* < 0.01) (Figure 1(B)). However, the testicular tissue from rats treated by LBSO showed that structure recovery as the normal form in spermatogenic cells appeared to be sequentially arranged in the lumen; this further substantiated that positive staining-integral optical density/area by β-galactosidase was increased significantly (*p* < 0.01) (Figure 1(B)) as compared to the ageing model, thereby validating the effects of LBSO on anti-ageing.

The ageing-related proteins, p16^INK4A^, p21^Waf1/Cip1^, p-*γ*H2AX, and β-galactosidase, presented a decreasing trend in the testicular tissue in the ageing model compared to the control group; this trend was reversed by LBSO treatment (all *p* < 0.001) (Figure 1(C–G)).

### LBSO alleviated oxidative stress-induced testis injury of ageing rats

**Figure 2.**
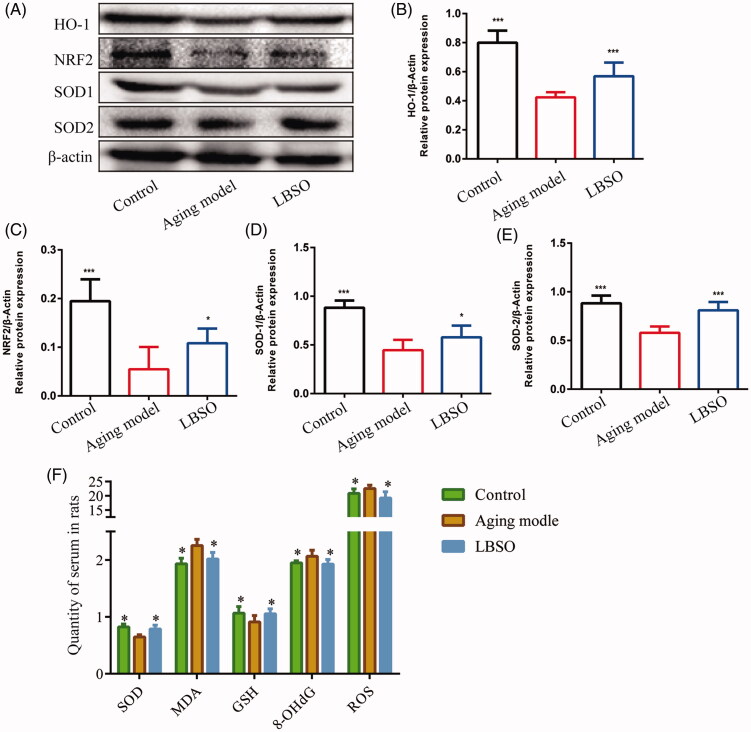
Effects of LBSO on antioxidative stress in the testis in *vivo*. (A) Representative Western blot image; mean densities of (B) HO-1, (C) NRF2, (D) SOD-1, and (E) SOD-2 in testicular tissue. Densitometry was used to compare the expression levels. β-Actin was used as an internal loading control. All data were expressed as the mean ± SD, *n* = 10; *p < 0.05, ***p < 0.001, compared with the ageing model. (F) The levels of SOD, 8-OHdG, ROS, MDA, and GSH in serum were measured. All data were expressed as the mean ± SD, *n* = 10; *p < 0.05, compared to the ageing model.

Compared to the ageing model of rats, the oxidation-related proteins, such as HO-1 (*p* < 0.05) (Figure 2(B)), NRF2 (*p* < 0.05) (Figure 2(C)), SOD-1 (*p* < 0.05) (Figure 2(D)), and SOD-2 (*p* < 0.05) (Figure 2(E)) presented an increasing trend in the testicular tissue of the LBSO group as compared, thereby presenting the effects of LBSO on antioxidative stress in the testis of ageing rats. In addition, a series of correlative factors, such as SOD, GSH, malondialdehyde (MDA), and 8-hydroxydeoxyguanosine (8-OHdG), were detected. Moreover, antioxidative factors, such as SOD (*p* < 0.05) and GSH (*p* < 0.05), significantly increased in the LBSO-treated rats as compared to the ageing model. Additionally, ROS (*p* < 0.05), MDA (*p* < 0.05), and 8-OHdG) were significantly declined (*p* < 0.05) in the serum of the LBSO group as compared to the ageing model (Figure 2(F)).

### LBSO improved the testicular function of ageing rats

**Figure 3.**
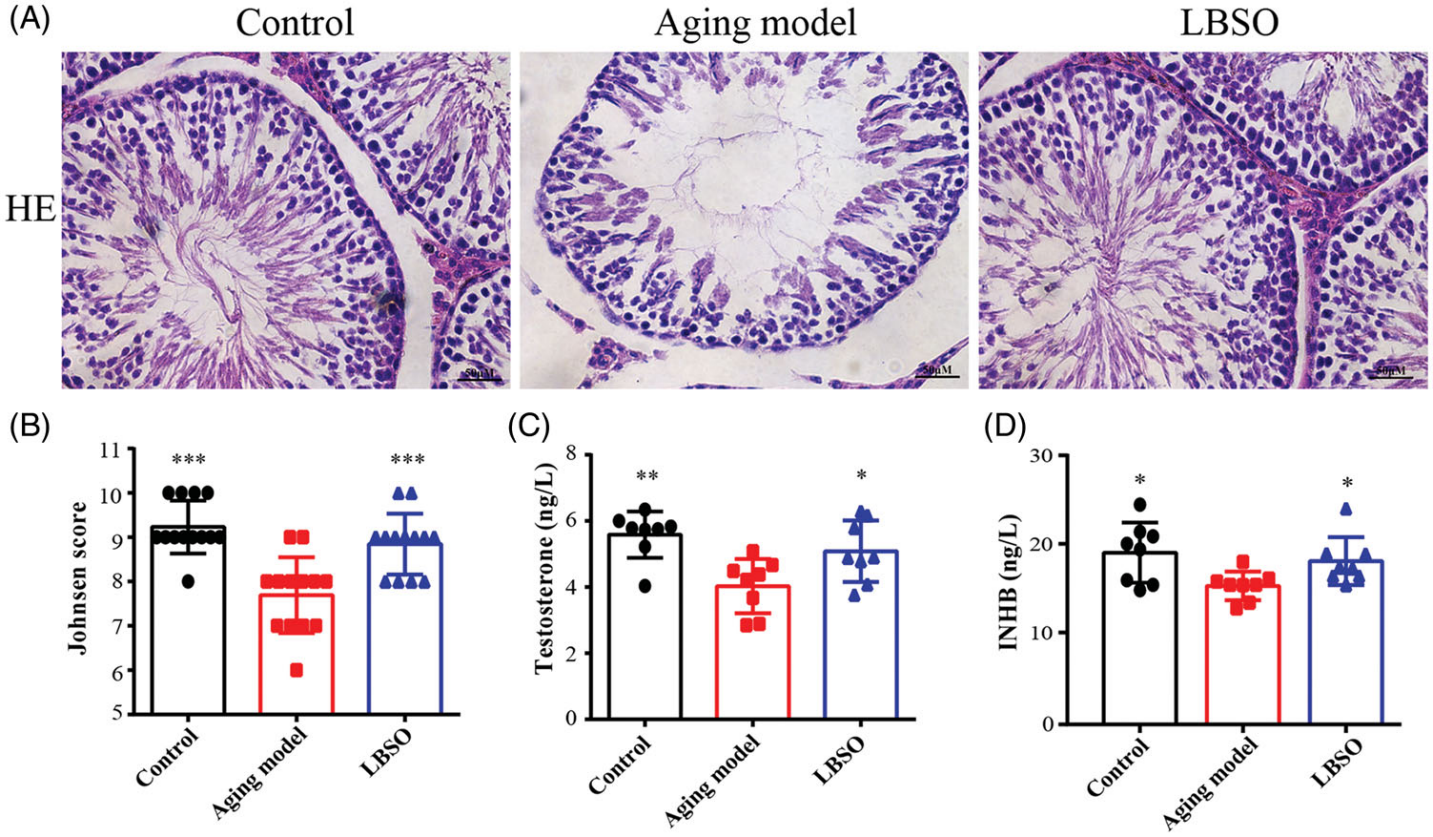
Function of testis was improved by LBSO. (A) Histomorphological changes in the testicular tissue of rats with LBSO. (B) Grades of testicular tissues depend on the Johnsen score. (C) Endocrine testosterone was detected in the rat serum. (D) Secretory cytokine level of INHB was measured in the rat serum.

The testicular tissue was assessed by Johnsen score to explore the spermatogenic function. Compared to the ageing model, the scores of LBSO raised considerably from 7.69 ± 0.24 to 8.85 ± 0.19, indicating that the testicular spermatogenic function was improved by treatment with LBSO (Figure 3(B)). Additionally, the INHB and testosterone, secreted by TM4 cells in serum were increased significantly in the LBSO group than in the controls (both *p* < 0.05) (Figure 3(C,D)).

### LBSO increased anti-ageing function on testes in ageing rats via SIRT3

**Figure 4.**
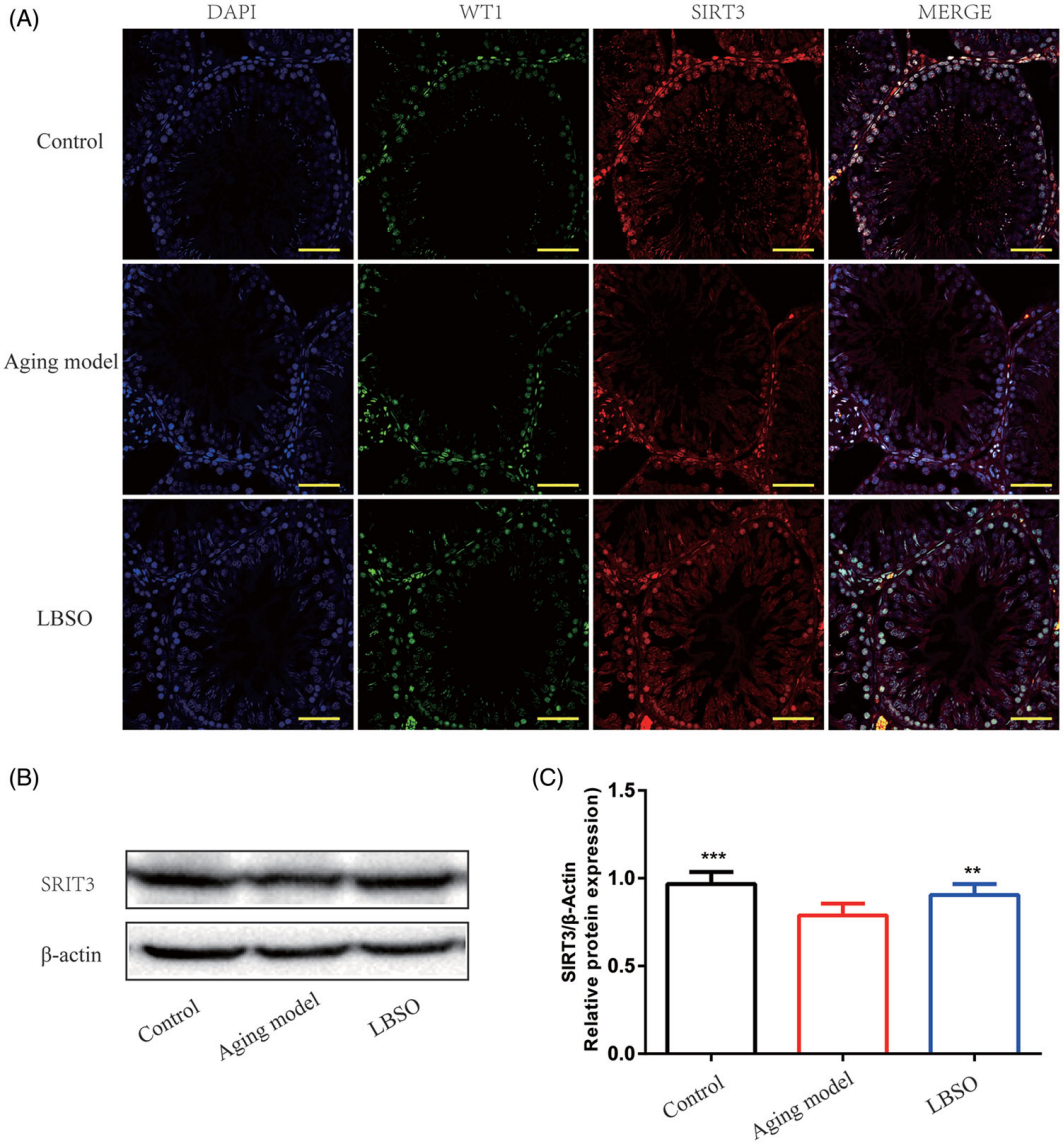
Expression SIRT3 in Sertoli cells in testicular tissue. (A) The location of Sertoli cells was labelled by specific marker WT1, and SIRT3 was localised in testicular tissue by IF. (B) Representative Western blot image of SIRT3. (C) The relative expression of SIRT3 in testicular tissue. Densitometry was used to compare the expression levels. β-Actin was used as an internal loading control. All data were expressed as the mean ± SD, *n* = 10; **p < 0.01, ***p < 0.001, compared to the ageing model.

In order to explore the role of LBSO in anti-ageing, the expression of SIRT3 was assessed and was found to be increased markedly in the testes of the LBSO group compared to the ageing model by Western blot (*p* < 0.01) (Figure 4(C)). As shown in Figure 4(A), SIRT3 was upregulated in the testicular tissue of rats administered LBSO intragastrically. IF of Sertoli cells showed increasing SIRT3 expression based on WT1 location in the TM4 cells.

### LBSO pre-treatment reduced oxidative stress injury in ageing TM4 cells

**Figure 5.**
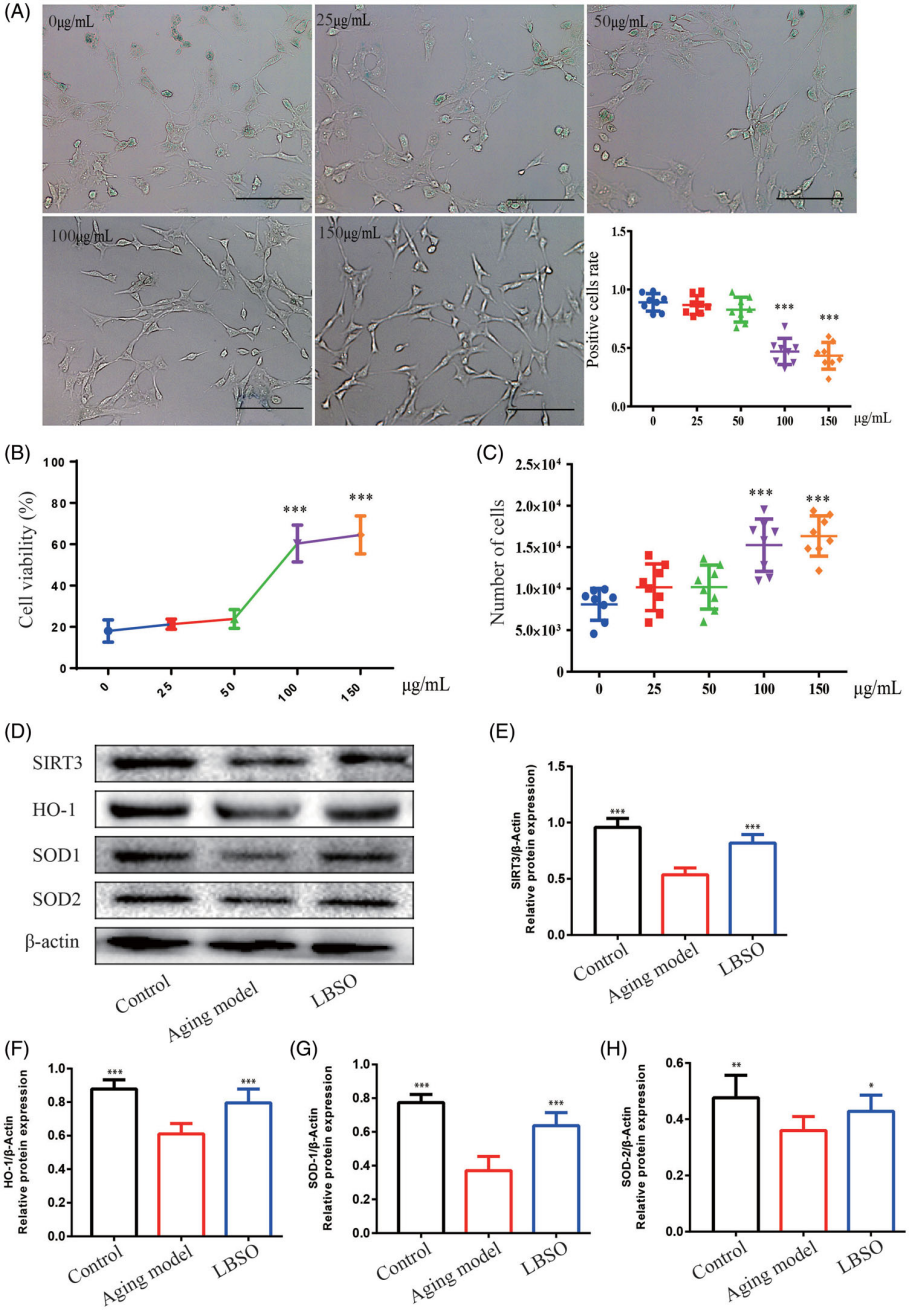
Optimal concentration of LBSO was screened *in vitro*. (A) TM4 cells were stained with β-galactosidase in the intervention of different concentrations of LBSO (magnification 100×), and the positive rate of cells in blue due to β-galactosidase. (B) The cell viability showed the ED_50_ of LBSO. (C) The number of TM cells was measured by CCK-8 in various concentrations of LBSO. (D) Representative Western blot image of SIRT3, HO-1, SOD-1, and SOD-2. (E–H) The relative expression of SIRT3, HO-1, SOD-1, and SOD-2 in testicular tissue. Densitometry was used to compare the expression levels. β-Actin was used as an internal loading control. All data were expressed as the mean ± SD, *n* = 10; *p < 0.05, ***p < 0.001, compared to the ageing model.

TM4 cells were treated by d-gal and used to sub-acutely mimic the ageing model. Subsequently, the ageing TM4 cells were pre-treated with different concentrations of LBSO and detected by β-galactosidase staining. The rate of positive cells (stained in blue) in the intervention of LBSO at 100 μg/mL (*p* < 0.001) and 150 μg/mL (*p* < 0.001) declined considerably as compared to ageing TM4 cells (Figure 5(A)). The cell viability was detected by CCK-8 kit, we found that ED_50_ of LBSO was 86.72 ± 1.49 (Figure 5(B)), calculating the number of cells observed to that intervened by 100 μg/mL (*p* < 0.001) and 150 μg/mL (*p* < 0.001) LBSO, which increased from 8120 ± 676.2 to 15251 ± 1119 and 16354 ± 858.1, respectively, albeit without statistical significance between the two concentrations (Figure 5(C)). In addition, SIRT3 (*p* < 0.001) (Figure 5(E)), HO-1 (*p* < 0.001) (Figure 5(F)), SOD-1 (*p* < 0.001) (Figure 5(G)), and SOD-2 (*p* < 0.05) (Figure 5(H)) protein levels were increased significantly in TM4 cells treated with LBSO at 100 μg/mL, compared with the ageing model.

### LBSO pre-treatment alleviated oxidative stress via SIRT3 pathway in ageing TM4 cells

**Figure 6.**
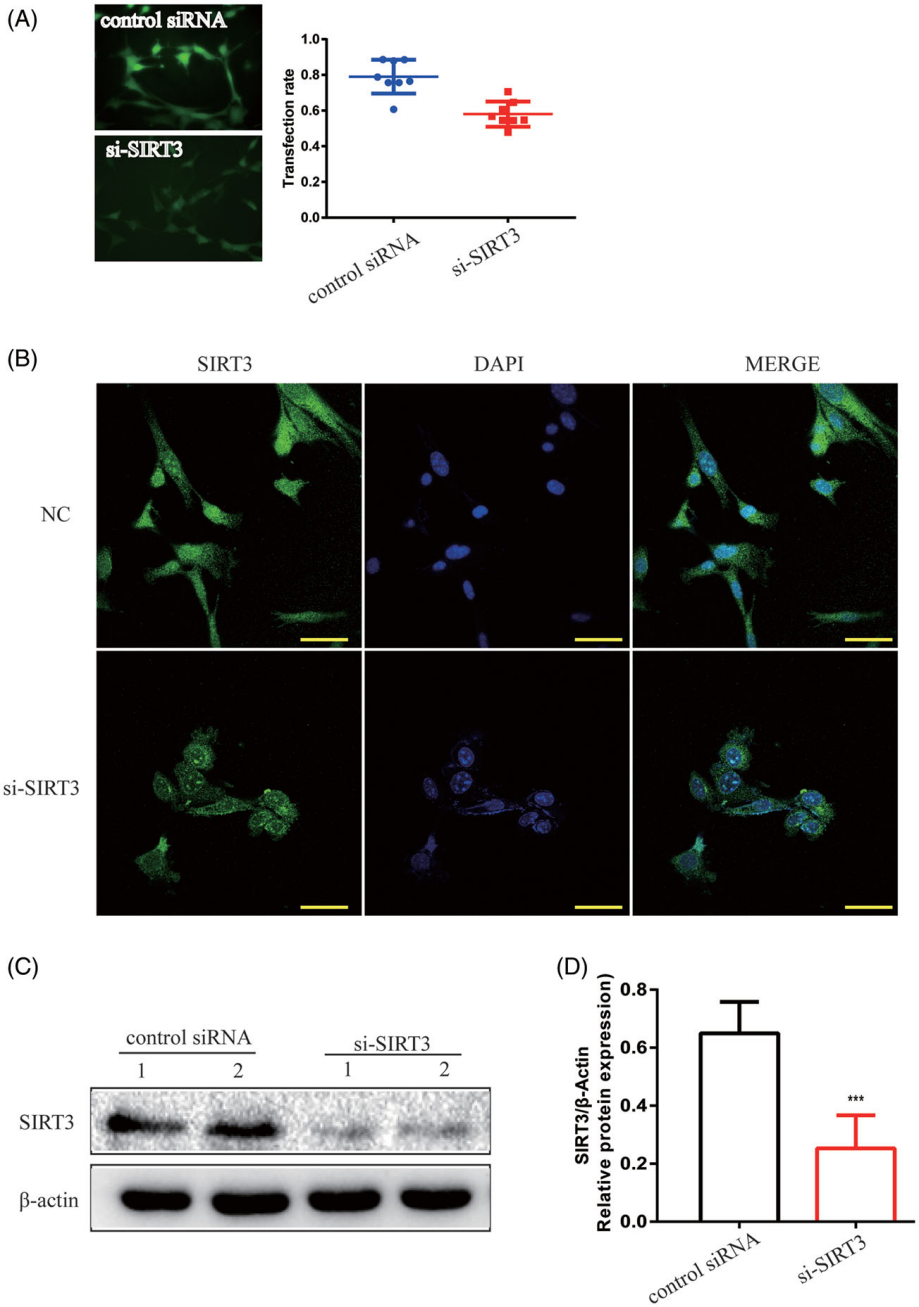
TM4 cells were infected by lentivirus with silencing SIRT3. (A) The transfection rate of cells was observed under a fluorescence microscope (magnification 200×). (B) The location of SIRT3 was expressed in TM4 cells by IF (magnification 400×). (C) The expression of SIRT3 in TM4 cells by Western blot. Densitometry was used to compare the expression levels. β-Actin was used as an internal loading control. Data were expressed as the mean ± SD, *n* = 8; ***p < 0.001, compared to the control siRNA group.

**Figure 7.**
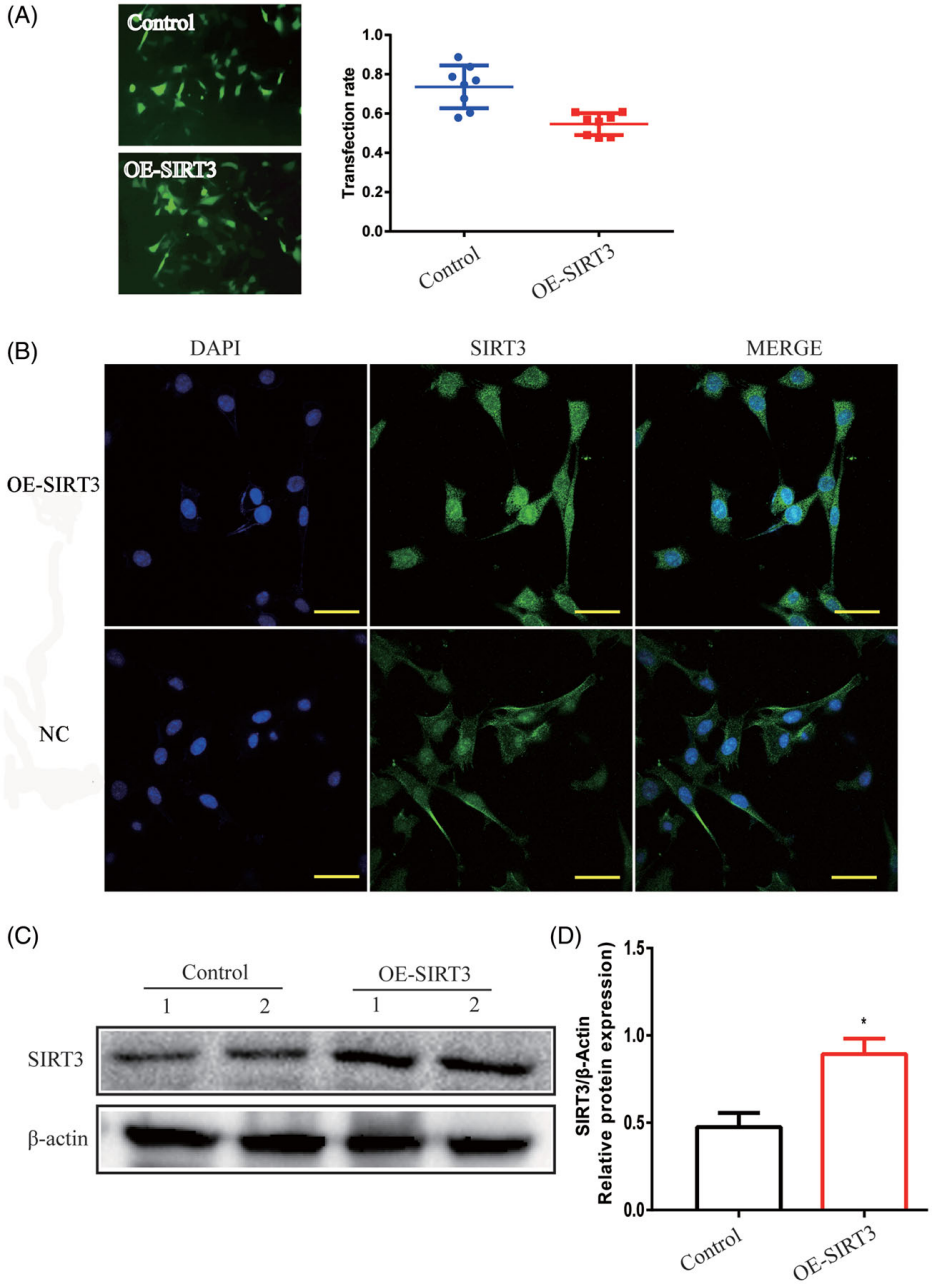
TM4 cells were transfected with SIRT3-overexpression- lentivirus. (A) The transfection rate of cells was observed under a fluorescence microscope (magnification 200×). (B) The location of SIRT3 in TM4 cells by IF (magnification 400×). (C) The expression of SIRT3 in TM4 cells by Western blot. Densitometry was used to compare expression levels. β-Actin was used as an internal loading control. All data were expressed as the mean ± SD, *n* = 8; *p < 0.05, compared to the control group.

TM4 cells were disposed when transfected by lentivirus to overexpress or silence SIRT3. The positive rates of transfection in SIRT3-overexpression and -silencing were 0.55 ± 0.020 and 0.58 ± 0.025, respectively, while the overexpression of SIRT3 protein presented the protein was upregulated significantly (*p* < 0.05) (Figure 6), while in SIRT3-silenced cells, the protein level was downregulated significantly (*p* < 0.001) (Figure 7) as compared to the control cells treated with lentivirus-NC.

**Figure 8.**
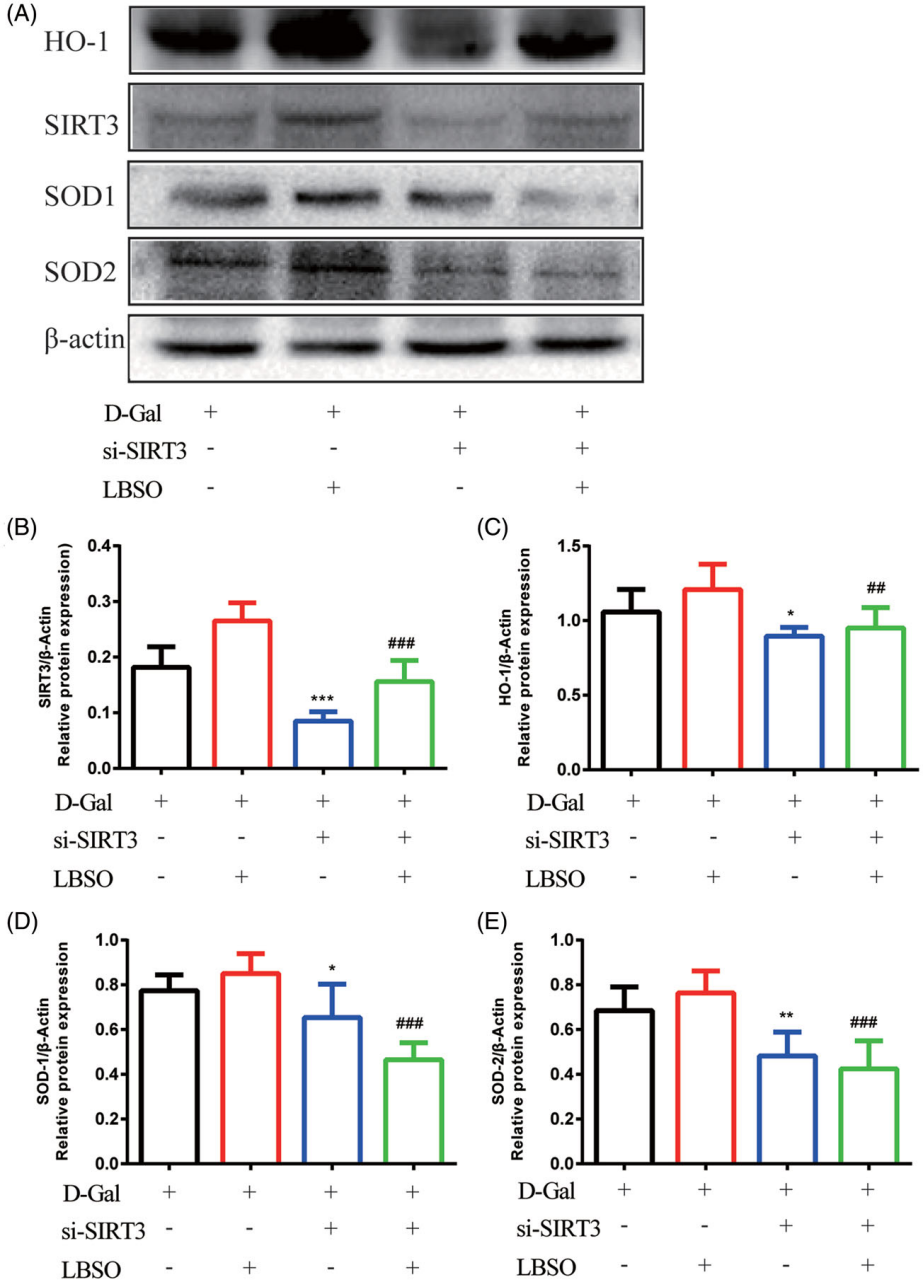
The effects of LBSO on anti-oxidation in SIRT3. (A) Representative Western blot image; mean densities of (B) SIRT3, (C) HO-1, (D) SOD-1, and (E) SOD-2 of TM4 cells in control, LBSO, si-SIRT3, and si-SIRT3-LBSO group. Densitometry was used to compare the expression levels. β-Actin was used as an internal loading control. All data are expressed as the mean ± SD, *n* = 10; *p < 0.05, **p < 0.01, ***p < 0.001, compared to the control group; ^##^*p* < 0.01, ^###^*p* < 0.001, compared to the LBSP group.

Furthermore, SIRT3-overexpressing or SIRT3-silenced cells were treated with LBSO before d-gal addition. SIRT3-silencing exhibited that TM4 significantly downregulated the expression of SIRT3 (*p* < 0.001), HO-1 (*p* < 0.05), SOD-1 (*p* < 0.05), and SOD-2 (*p* < 0.01) as compared to the control group. Despite SIRT3-silenced cells intervened by LBSO (si-SIRT3-LBSO group), the expression of SIRT3 (*p* < 0.001), HO-1 (*p* < 0.01), SOD-1 (*p* < 0.001), and SOD-2 (*p* < 0.001) declined as compared to the LBSO group (Figure 8).

**Figure 9.**
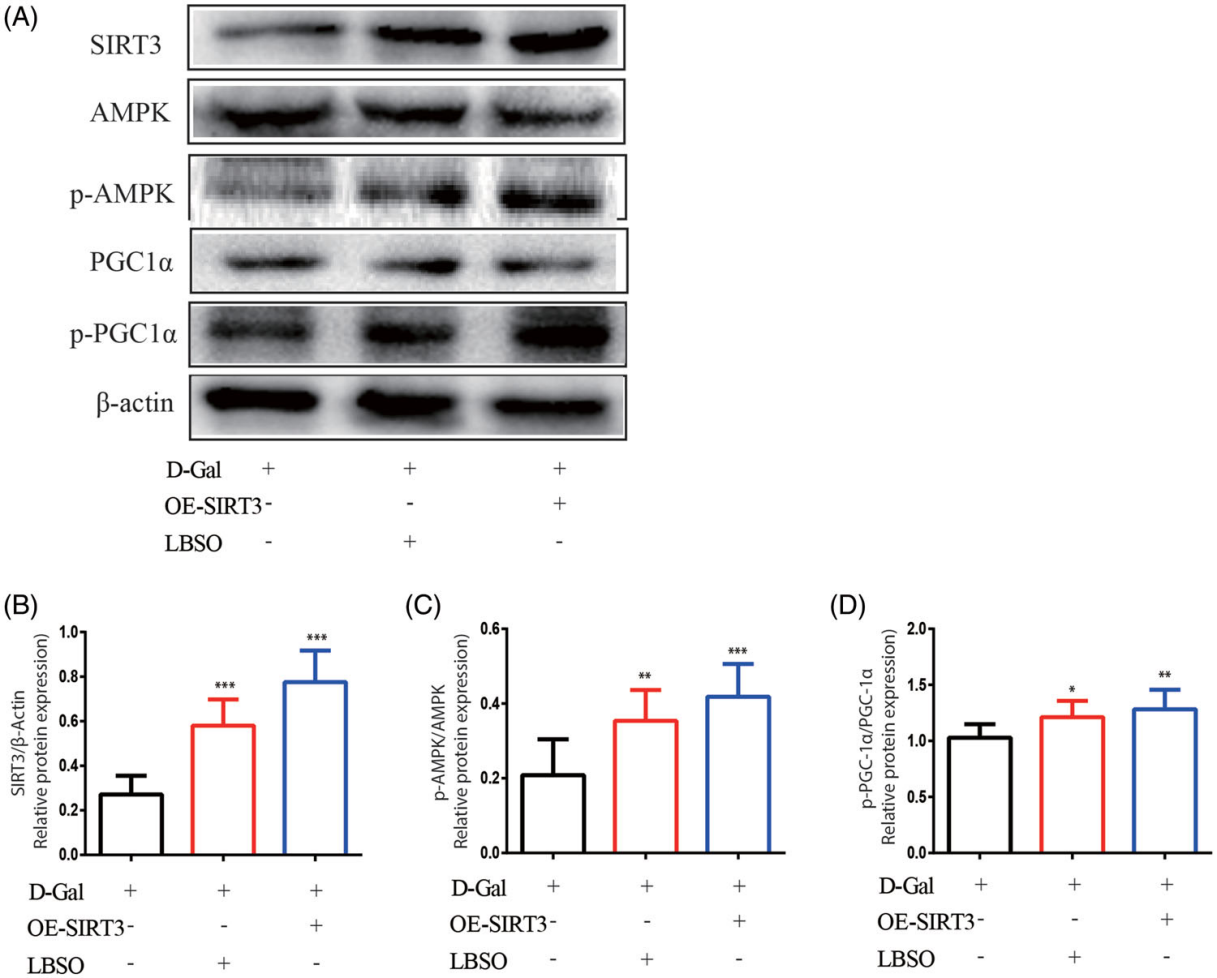
The effects of LBSO on anti-oxidation via SIRT3/AMPK/PGC-1α. (A) Representative Western blot image; mean densities of (B) SIRT3, (C) AMPK, (D) SOD-1, and (E) PGC-1α of TM4 cells in the control group, OE-SIRT3 group, and LBSO group. Densitometry was used to compare the expression levels. The data are expressed as the mean ± SD, *n* = 10; *p < 0.05, **p < 0.01, ***p < 0.001, compared to the control group.

Figure 9 showed that the expression of SIRT3 (*p* < 0.001) increased significantly in both SIRT3-overexpressing and LBSO-treated TM4 cells compared to the control group. In addition, the expression of p-AMPK/AMPK (*p* < 0.001) and p-PGC-1*α*/PGC-1*α* (*p* < 0.01) in SIRT3-overexpressing TM4 cells upregulated significantly, and both p-AMPK/AMPK (*p* < 0.01) and p-PGC-1*α*/PGC-1*α* (*p* < 0.05) were increased considerably in cells treated with LBSO compared to the control group.

## Discussion

The present study aimed to investigate the effects of LBSO on antioxidative stress by activating SIRT3, with LBSO improving the tolerance of TM4 cells in the intervention of d-gal while TM4 cells with si-SIRT3 failing to resist the circumstance of d-gal. However, the functions of LBSO were explored to find a new approach to protect the testis from ageing and improve the function of the testis.

In the present study, LBSO successfully alleviated oxidative stress in ageing testis triggered by upregulating NRF2 and its downstream HO-1, eliminating ROS and MDA accumulation in cells, and improving the capacity of antioxidation by raising the level of SOD, GSH, and 8-OHdG. These phenomena proved that the LBSO administration has antioxidative capacity in testicular tissue via NRF2/HO-1. *SIRT3*, a downstream gene of the NRF2 signalling pathway, had been proven to have a role in the longevity program (Satterstrom et al. 2015). The concept of ageing in a time-dependent accumulation of cellular oxidative damage is at the core of the ageing process (Höhn et al. 2017), which is vital to protect the cells from oxidative stress to postpone the ageing process. In recent years, proteomic methods have shown that SIRT3 regulates the acetylation of the lysine amino acid residues in mitochondria, which, in turn, maintains mitochondrial homeostasis (Ciregia 2019). In addition, the regulation of ROS and ROS-induced damage by SIRT3 is a critical mechanism by which SIRT3 influences ageing and the diseases related to ageing (Park et al. 2011; McDonnell et al. 2015). The present study showed the upregulation of SIRT3 expression in testicular tissue after LBSO administration in Sertoli cells. In order to validate the function of the testis, the related factors, such as INHB and testosterone secreted by Sertoli cells, could be the hallmarks to assess the secretion function of the testis (Chong et al. 2017). Moreover, the grades of sperms and spermatocytes in the different phases in the testicular tissue were marked by the Johnsen score. Subsequently, we found that the grades of Johnsen score and levels of INHB and testosterone in serum increased in the LBSO group compared to the ageing model, rendering that LBSO improved the testis function via antioxidation.

In the present study, TM4 cells were treated with lentivirus to silence SIRT3; as a result, the antioxidative capacity was dropped by declined expression of HO-1, SOD-1, and SOD-2. This indicated that SIRT3-silenced TM4 cells, exposed to d-gal, failed to tolerate this stimulation and severely damaged mitochondrial homeostasis, although SIRT3-silenced cells pre-treated with LBSO also failed to alleviate the oxidative stress caused by d-gal. Together, these results showed that SIRT3 was transported into the mitochondrial matrix and then activated through proteolytic processing at the N-terminus. It deacetylates many enzymes involved in response to oxidative stress and mitochondrial integrity (Salvatori et al. 2017; Yi et al. 2019). Therefore, we deduced that LBSO relieves oxidative stress using SIRT3.

The cellular deterioration seen with increasing age is caused by ROS, produced especially by the mitochondria (Giorgi et al. 2018). The current study suggested that LBSO relieved oxidative stress in d-gal-induced ageing testes by activating SIRT3 and further protected the mitochondria from impairment via AMPK/PGC-1α. Several studies showed that the mechanisms underlying extended lifespan or stress resistance are dependent on AMPK by compensating for the energy deficit and increasing the ability of antioxidative stress. Further, activated AMPK initiates PGC-1α, which regulates mitochondrial oxidative metabolism to protect the cells against ageing (Burkewitz et al. 2014; Ruiz et al. 2016). These findings implied that LBSO is a potential supplement for anti-ageing. AMPK is a crucial molecule in the cells regulating bioenergy metabolism, promoting longevity by regulating energy and nutrients in the cells (Salminen and Kaarniranta 2012). PGC-1α is a transcriptional coactivator that binds to and co-activates transcription factors, leading to the coordinated regulation of mitochondria and nuclear-encoded mitochondrial enzymes (Villena 2015). The activation of AMPK plays a critical role in the ageing process, usually determined by the ratio of p-AMPK/AMPK (Krishan et al. 2020), involved in the regulation of a series of ageing-related signalling pathways, such as SIRT1 (Chen et al. 2019), CRTC-1 (Mair et al. 2011), and PGC-1α (Kou et al. 2018). Activated AMPK increased the intracellular NAD+, affecting the activity of PGC-1α through phosphorylation and deacetylation (McDonnell et al. 2015). Finally, the findings suggested that the administration of LBSO alleviated oxidative stress in ageing TM4 cells by regulating AMPK/PGC-1α-mediated signal transduction. We found that TM4 cells treated by overexpression of SIRT3 or LBSO restored the expression of AMPK and PGC-1α in the presence of d-gal. This indicated that the invention of LBSO is valid for upregulating AMPK/PGC-1α-mediated signal transduction to protect TM4 cells against d-gal-induced oxidative injury concurrent to SIRT3 overexpression. Therefore, we deduced that LBSO activates SIRT3 against oxidative stress in ageing TM4 cells, wherein downstream AMPK/PGC-1α was successfully was activated and participated in the regulation of oxidative injury repair.

However, the limitation of this study was obvious, it is difficult to show the LD_50_ of LBSO, since rats received the maximal dosage of LBSO where rats still survived, as well as the LBSO is hard to dissolve into culture medium where we couldn’t detect its LD_50_. It may attribute into the security of LBSO which is an ideal supplement not a drug.

## Conclusions

LBSO exerts its potential function on the antioxidative stress in the testis *in vivo* and *in vitro*. This phenomenon involves the SIRT3 signalling pathway. In light of these results, it seems possible to affirm that LBSO alleviated oxidative stress in d-gal-induced sub-acutely ageing testis and TM4 cells. The mechanism implied that LBSO suppressed the oxidative stress to mitochondria in TM4 cells via SIRT3/AMPK/PGC-1α. However, to exploit the potential as a supplement to preserve fertility in males, future studies would investigate the effects of LBSO on improving the functions of the testis. Thus, this analysis provides a platform for future studies to investigate the effects of LBSO on ageing.
